# The Wolf Hidden behind the Clots: Catastrophic Antiphospholipid Antibody Syndrome

**DOI:** 10.1155/2018/4693037

**Published:** 2018-07-19

**Authors:** Myat Han Soe, Krishna Adit Agarwal, Alueshima Akough-Weir

**Affiliations:** Department of Medicine, Baystate Medical Center, University of Massachusetts Medical School, Springfield, MA, USA

## Abstract

Catastrophic antiphospholipid syndrome (CAPS) is a rare but highly fatal clinical syndrome that occurs in up to 1% of patients with antiphospholipid syndrome (APS). The diagnosis of CAPS is often delayed because its presentation with multiple organ thromboses can be confused with other thrombotic microangiopathies and severe sepsis. We report a case of CAPS in a patient with APS and systemic lupus erythematosus (SLE) presenting with thrombotic storm precipitated by trauma, cytomegalovirus (CMV) infection, and noncompliance with anticoagulation therapy. Our case reflects the “two-hit hypothesis” of APS in which the presence of antiphospholipid antibodies (first hit) increases the thrombophilic risk, and thromboses take place in the presence of another thrombophilic condition such as CMV infection in our case. In this case review, we discuss the diagnostic challenges and management of CAPS. In clinical practice, we aim to stress the importance of thorough evaluation and management of precipitating events such as infections in addition to timely diagnosis and treatment of this catastrophic clinical entity.

## 1. Introduction

Antiphospholipid antibody syndrome (APS) is a multisystem autoimmune disorder characterized by vascular thromboses and/or pregnancy loss associated with persistently positive antiphospholipid antibodies (aPL) indicated by lupus anticoagulant (LA) test, anticardiolipin antibodies (aCL), and/or anti-beta2 glycoprotein-I antibody (aB2GPI) [1]. This disorder is referred to as primary when it occurs in the absence of other autoimmune diseases, while the secondary APS occurs in the setting of an autoimmune disorder such as systemic lupus erythematosus (SLE) [2]. In its most severe form, patients develop multiple organ small vessel thromboses, a life-threatening condition referred to as catastrophic APS (CAPS). This occurs in up to 1% of patients with APS [3]. The unique features of CAPS include (a) a rapid onset of thromboses with resultant multiple organ dysfunction, (b) common association with other thrombotic microangiopathies (TMAs), (c) evidence of systemic inflammatory response syndrome (SIRS), which can mimic sepsis, (d) high risk of unusual organ involvement, and (e) relatively high mortality rate albeit optimal therapy [1]. As most patients with circulating aPL (first hit) do not develop thromboses, an exposure to a second hit such as infection, malignancy, trauma, or surgical procedure has been proposed as a precipitating factor to induce the thrombotic event [4]. One analysis has shown that 40% of APS cases (*n*=100) developed CAPS after infectious episodes like skin infections (18%), human immunodeficiency virus (HIV) infection (17%), pneumonia (14%), hepatitis C virus (HCV) infection (13%), and urinary tract infection (10%) [5]. CAPS precipitated by CMV infection is rare, and only 3 cases have been identified in literature [5]. We report the fourth case of CAPS in a patient with SLE and APS presenting with high fever and acute multiorgan thromboses due to thrombotic storm precipitated by concomitant CMV infection.

## 2. Case Description

A 22-year-old Hispanic female with history of deep vein thrombosis (DVT) and pulmonary embolism (PE) at the age of 16, followed by diagnosis of SLE, acquired protein S deficiency and secondary APS, failed anticoagulation with Coumadin and enoxaparin due to noncompliance, status post inferior vena cava (IVC) filter placement, and currently on fondaparinux and chronic prednisone (20 mg) presented with generalized weakness, malaise, recurrent fevers, and elevated blood pressure. The patient had a road traffic accident and a viral upper respiratory tract infection diagnosed one week before this admission. She was not compliant with her medications including fondaparinux at this presentation. Clinical assessment revealed a fever of 101.3-degree Fahrenheit, blood pressure of 140/115 mmHg with tachycardia up to 130 s, anemia with hemoglobin of 6.5 gm/dl, and acute kidney injury with creatinine of 1.4 mg/dl and ESR of 95.

**Figure 1:**
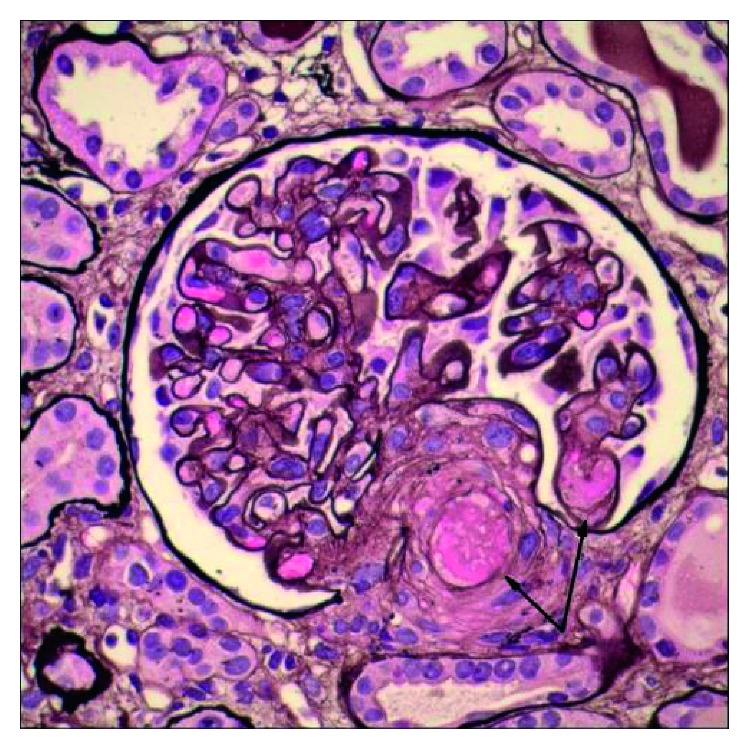
Light microscopy image showing a glomerular tuft with capillary thrombus and an arteriolar thrombus at vascular pole (arrows). Stain: Jones methenamine. Magnification: 600x.

**Figure 2:**
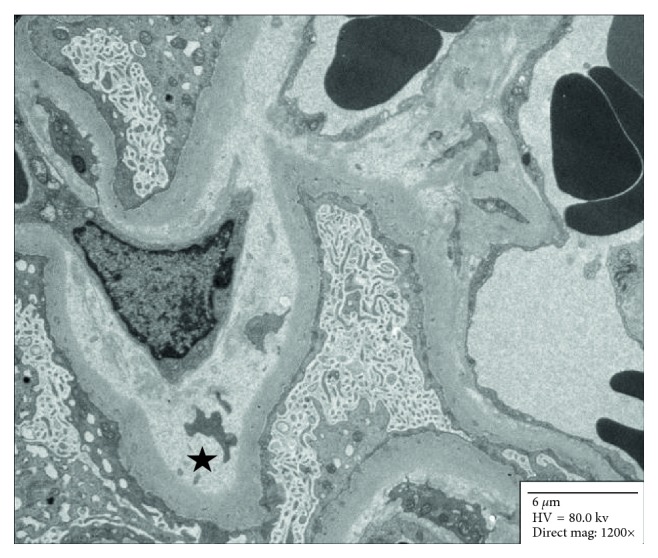
Electron microscopy image showing a glomerular capillary loop with subendothelial widening and lucency (star), typical of microangiopathic endothelial cell injury. Magnification: 1200x.

The patient was treated with broad-spectrum antibiotics for possible infection due to the presence of fever, tachycardia, and leukocytosis, concerning for sepsis. However, her symptoms did not subside with antibiotic treatment. Renal function continued to decline, and hemoglobin continued to drop along with worsening thrombocytopenia requiring multiple units of blood transfusion. She developed livedo reticularis, right upper extremity weakness, memory loss, cyanotic left toes with diminished bilateral dorsalis pedis pulses, and absent right radial pulse. The arterial Doppler study revealed absence of flow in the distal right radial artery. MRI brain was consistent with multifocal embolic stroke. Echocardiogram to evaluate for cardioembolic etiology revealed no thrombus but a new mitral regurgitation (MR). Incidentally, she was also found to have splenic infarcts. Her clinical scenario was consistent with widespread embolization or thromboses with end-organ damage. Blood cultures were negative and echocardiogram revealed no vegetation. Therefore, the etiology was unlikely to be infective endocarditis or sepsis. Disseminated intravascular coagulation (DIC) and thrombotic thrombocytopenic purpura (TTP) were excluded by the lack of significant schistocytosis on a peripheral blood review and normal ADAMTS13 activity. Immunology workup revealed low complement C3 and C4 with high CH50, high titer of anti-double stranded DNA (dsDNA) antibody, positive LA, positive aCL, and negative aB2GPI. Infection screen for bacterial and viral etiology including syphilis, HIV, and HCV was negative except for positive CMV PCR with a viral load of 2300 IU/ml. Renal biopsy showed class II lupus nephritis and thrombotic microangiopathy with glomerular capillary and arteriolar thromboses, consistent with APS (Figures 1 and 2).

Given her history of APS with the described workup profile, diagnosis of CAPS was made and concurrent CMV infection was identified as the precipitating culprit. Anticoagulation with intravenous heparin was initiated for widespread thromboses despite the presence of thrombocytopenia. The patient also received pulsed intravenous methylprednisone. Plasmapheresis was initiated followed by intravenous immunoglobulin (IVIG) for 5 days (400 mg/kg body weight daily) due to lack of significant clinical improvement. Mycophenolate and hydroxychloroquine were used for class II lupus nephritis. Due to immunosuppression, CMV infection was treated with valganciclovir for about 4 weeks till viral load became undetectable. With gradual recovery, anti-dsDNA antibody, aPL as well as aCL titers decreased, and complement levels normalized with resolution of skin lesions, mitral regurgitation, AKI, anemia, and thrombocytopenia. She was transitioned to enoxaparin (60 mg twice daily) on discharge and continued on prednisone (60 mg daily), mycophenolate (1000 mg twice daily), and hydroxychloroquine (200 mg twice daily). Fondaparinux was not continued because of acute kidney injury. Over the course of the following one year, her prednisone was tapered. She remained free of recurrent thrombotic events.

## 3. Discussion

CAPS is a rare life-threatening form of APS in which thrombotic angiopathy results in multiple organ dysfunction [2]. Diagnosis of CAPS can be missed due to lack of awareness of CAPS in APS patients, diagnostic criteria, and precipitating events. As CAPS carries a high mortality, timely diagnosis and treatment are of paramount importance. As per the International Consensus Guidelines, the definite diagnosis of CAPS requires four criteria to be fulfilled: (1) involvement of three or more organs/tissues, (2) development of manifestations simultaneously or within one week, (3) histologic evidence of vascular thrombosis, and (4) laboratory confirmation of presence of antiphospholipid antibodies [6].

Clinical manifestations of CAPS with multiple organs' involvement are usually secondary to acute thrombotic microangiopathy with resultant microangiopathic hemolytic anemia and thrombocytopenia [2]. This can be mistaken with other TMAs such as TTP and HUS. However, ADAMTS13 level is normal in CAPS and fewer schistocytes are seen in CAPS compared to TTP and HUS [2]. Regarding organ involvement, the kidneys, lungs, CNS, heart, skin, liver, and GIT are the most commonly affected organs in CAPS. Renal involvement can be as high as 73% with the most frequent manifestations being hypertension, proteinuria, which can be in nephrotic range, hematuria, and acute renal failure [7]. In our patient, renal manifestations included hypertension, hematuria, and AKI. Pulmonary involvement ranges from 24% on presentation, increasing up to 64% during the course of disease. Manifestations can include ARDS, diffuse alveolar hemorrhage, and pulmonary embolism [2]. Cardiac manifestations can be present in 10% of cases on presentation, increasing up to 51% during the course of disease. These include MI and valvular heart disease such as MR, Libman–Sacks endocarditis, and arrhythmias [2].

In majority of patients, a precipitating factor can be identified, including infection (22%), surgery (10%), discontinuation of anticoagulation (8%), medications (7%), obstetric complications (7%), and malignancy (5%) [2]. One study showed that CAPS developed in 40% of APS patients after infectious episodes [5]. Two-hit hypothesis has been suggested to explain the development of CAPS in APS patients after a precipitating event. aPL might be persistently present but the catastrophic events occur only occasionally as aPL antibodies per se increase the thrombophilic risk but are not thrombogenic [8]. It is postulated that infections and other precipitating factors described above cause a conformational change in circular/nonantigenic beta2GPI towards open/oxidized form exposing its antigenic domain I. This conformational change allows aPL interaction with antigenic domain I of beta2GPI triggering further production of aPL, endothelial activation, and propagation of platelet activation with subsequent thrombosis [9]. Consequently, the catastrophic event develops with multiorgan thromboses.

CMV infection itself was described to be associated with mesenteric and femoropopliteal thrombosis in a case reported by Labarca [10]. Another case report described that a patient developed transient APS during the course of CMV infection [11]. Gharavi et al. demonstrated that synthetic peptides which share a structural homology with the putative phospholipid-binding domain of beta2GPI and CMV were able to induce aPL in animal studies. Their results indicate that aPL induced by immunization with phospholipid-binding CMV peptides are pathogenic in vivo [12, 13]. Our case describes CMV infection as the precipitating event for CAPS. Interestingly, aB2GPI IgG and IgM were negative (<4 U/ml) during the catastrophic event precipitated by concurrent CMV infection and became high after resolution of CMV infection. This makes our case unique as compared to the other CMV-triggered CAPS cases in which fluctuations in aB2GPI were not described. Based on the studies described above, we postulate that CMV peptides which share the structural homology with phospholipid binding region of B2GPI might have bound to aB2GPI so that the assay could not detect free antibodies.

Therapeutic approach involving intensive anticoagulation, immunosuppression, and treatment of precipitating event such as infection and associated conditions such as SLE is the mainstay of treatment for CAPS. Thrombotic events are treated with full anticoagulation with intravenous or subcutaneous heparin followed by warfarin with INR target of 2 to 3 for venous thromboses and 3 for arterial thromboses. For patients with recurrent thromboses, INR should be targeted between 3 and 4. Rituximab can be used for recurrent thromboses despite adequate anticoagulation [14]. There are no existing data regarding the role of newer oral anticoagulants in CAPS yet. Immunosuppression is usually achieved with use of IV-pulsed methylprednisone and elimination of preexisting antibodies with plasmapheresis and IVIG. A small series of study of patients with CAPS concluded that those who received triple therapy with anticoagulation, steroids, and either plasma exchange, IVIG, or both had significantly lower mortality rates compared with those treated without plasma exchange, IVIG, or both [15]. Glucocorticoids reduce the inflammatory cytokine storm triggered by multiple thromboses and ischemic tissues. Precipitating infection, if identified, must also be treated aggressively. In our case, CMV infection was treated with valganciclovir till viral load was undetectable given the concomitant use of immunosuppressants. In patients with underlying SLE, immunosuppressants such as mycophenolate and cyclophosphamide can be used, especially if CAPS is not responding well to steroid and IVIG/plasmapheresis [16].

## 4. Conclusion

CAPS is a rare form of APS with a high mortality rate of about 50% [17]. The main causes of death described in the “CAPS Registry” were infection (20%), stroke (19%), cardiac failure (17%), and multiorgan failure (17%) [18]. Clinicians should be aware of the existence of this catastrophic disease along with its diagnostic criteria and precipitating factors. We should consider CAPS in the differential diagnosis of APS patients presenting with high fever and evidence of end-organ damage because timely diagnosis and treatment are crucial for management of CAPS. Early anticoagulation is of particular importance despite the presence of severe thrombocytopenia. Unfractionated heparin infusion is preferred over low-molecular-weight heparin in CAPS [16]. IVIG alone is preferred over plasmapheresis-IVIG combination in the presence of hemodynamic instability. High-dose steroid is the first line of treatment for CAPS. Precipitating factors must be addressed accordingly. Immunosuppressants such as cyclophosphamide can be used in CAPS not responding well to steroid treatment or in patients with underlying SLE. A prognostic study showed that up to 66% of the patients who survive the initial catastrophic event remained symptom-free with anticoagulation during an average follow-up period of 67.2 months [19].
